# Nuclear medicine imaging in non-seminomatous germ cell tumors: lessons learned from the past failures

**DOI:** 10.1186/s40644-024-00794-5

**Published:** 2024-11-18

**Authors:** Narjess Ayati, Emran Askari, Maryam Fotouhi, Masume Soltanabadi, Atena Aghaee, Hesamoddin Roustaei, Andrew M. Scott

**Affiliations:** 1 Department of Theranostics and Nuclear Medicine, St. Vincent’s Hospital, Sydney, NSW Australia; 2 St. Vincent’s Clinical School, University of New South Wales, Sydney, NSW Australia; 3 Nuclear Medicine Research Center, Mashhad University of Medical Sciences (MUMS), Mashhad, Iran; 4 Advanced Diagnostic and Interventional Radiology Research Center (ADIR), Cancer Institute, Imam Khomeini Hospital Complex, Tehran University of Medical Sciences, Tehran, Iran; 5 Rajaie Cardiovascular Medical and Research Center, Iran University of Medical Sciences, Tehran, Iran; 6 Department of Nuclear Medicine, University Hospital Salzburg, Paracelsus Medical University, Salzburg, Austria; 7 Tumour Targeting Laboratory, Olivia Newton-John Cancer Research Institute, Melbourne, VIC Australia; 8 School of Cancer Medicine, La Trobe University, Melbourne, VIC Australia; 9 Department of Molecular Imaging & Therapy, Austin Health, 145 Studley Road, Heidelberg, VIC 3084 Australia; 10 Department of Medicine, University of Melbourne, Melbourne, VIC Australia

**Keywords:** Non-seminomatous germ cell tumor, Teratoma, Retroperitoneal residual mass, Radiotracer, Imaging

## Abstract

There is an unmet need for a more accurate molecular imaging radiotracer in the field of non-seminomatous germ cell tumors (NSGCT). The clinical problem is that no single imaging modality is able to differentiate teratoma from necrotic tissue in NSGCTs, which the nuclear medicine techniques are no exception. The exponential growth in the list of potentially promising radiotracers may hold promise in the future for imaging of NSGCTs. Here, we have reviewed the past efforts and potential future advances in this field.

## Epidemiology

Testicular cancer is the most common malignancy in men aged 15–45 years and accounts for 1-1.8% of all male cancers [1, 2]. Of testicular malignancies, 95% are germ cell tumors (GCTs) [3]. About 50% of patients with GCT present with advanced non-seminomatous germ cell tumors (NSGCT) [4, 5].

## The imaging problem in the field of GCT

Following radical orchiectomy and adjuvant cisplatin-based triplet chemotherapy for the treatment of stage IIB-III NSGCT, there is a 30–40% chance of retroperitoneal mass persistence [6–9]. In 40–51% of these cases the retroperitoneal masses represent necrotic/fibrotic tissues, while 30–47% are teratomas, and the remaining 6–17% of cases are different histopathologies simply grouped as viable GCTs [10–12]. Post-chemotherapy retroperitoneal lymph node dissection (PC-RPLND) aims to eradicate all the remaining viable malignant tissue in the advanced NSGCTs, however distinguishing residual viable tumor from post-therapy changes remains a major challenge when deciding if surgery is required [13].

Several studies have shown that not only the viable tumors but also those with teratoma are at increased risk of recurrence [14–18]. For instance, Nestler and colleagues [14] analyzed data from a multi-center cohort of 1204 non-seminomas who underwent PC-RPLND and observed a significantly increased risk of recurrence by five years in the viable GCT/teratoma subgroups compared to patients with only necrosis (81% and 59%, vs. 19%, respectively, *p* < .001). Moreover, teratomas should be resected due to their resistance to chemoradiation, compressive effect on adjacent organs, and their ability to undergo malignant transformation, especially in the subtype of teratoma with somatic malignancy [11, 19]. Therefore, differentiation of teratomas from necrosis/fibrosis is clinically relevant.

Retroperitoneal teratomas are usually asymptomatic, and tumor markers frequently fall within the normal range, except in cases of mixed GCT or those with mucinous or hepatoid differentiation. Therefore, its detection and follow-up are highly reliant on anatomic imaging [19, 20]. Mature teratomas, also referred to as differentiated teratomas, usually present as low attenuation retroperitoneal masses with less aggressive behavior [21]. There are some conventional imaging features that are useful for the differentiation of mature teratoma from immature or growing teratoma (Table 1). However, irrespective of their subtype, teratomas should be resected as per international guidelines [22, 23].

**Table 1:** Anatomic features for differentiation and prognostication of mature teratoma versus immature or growing teratoma across a prognosis range from poor to good [20, 22, 109]

Radiologic feature	Prognostic Impression	
	Poor	Good
Echogenicity	Solid	Cystic with heteroechoic and hyperechogenic foci ^*1^; onion ring appearance ^*2^
Vascularity	Hypervascular	Hypovascular
Borders	Indistinct	Distinct
Contrast enhancement	Heterogeneous enhancement ^*3^	Mild
Nodular formation	Yes	No
New lesions or increase in size of previous lesions	Frequent	Infrequent
Location	Retroperitoneal, mediastinal and intracranial	Confined to retroperitoneum
Spontaneous regression	Rare	Reported in burnout teratomas

^*1^ Mature teratomas may eventually grow in previous sites of metastasis, presenting as cystic changes with heterogeneous density changes containing calcification and fat. In these cases, serial follow-up CT imaging may be indicated before proceeding to surgery

^*2^ Associated with dermoid cysts

^*3^ For contrast-enhanced CT, solid portion of the mass along with septations are enhanced while the cystic fat-containing component usually remains unchanged

## Review of the guidelines

Unfortunately, no non-invasive diagnostic modality or validated risk calculator can accurately determine the nature of the residual mass (Table 2) [6, 11, 13, 24–29]. Therefore, the EAU guidelines recommends resecting the post-chemotherapy residual mass if > 1 cm in greatest diameter on contrast-enhanced CT (ceCT) whenever feasible [30]. In this context, PC-RPLND serves as both a diagnostic and a therapeutic tool [28]. This approach will over-treat almost half of the patients while leaving 25% risk of teratoma and 5% risk of viable tumor in small sub-centimetric lesions, which have an overall 6–9% risk of relapse and may be captured by subsequent imaging [3, 11, 27]. In this context, surgery is often without oncological benefit [14] and major post-surgical complication rates are non-negligible according to a systematic review [31].

**Table 2:** Prognosticators of post-chemotherapy retroperitoneal residual mass

Favorable prognosis	Unfavorable prognosis
No teratoma component in the orchiectomy specimen [27] ^*1, *2^	Abnormal tumor markers [53]
Normal pre-chemotherapy AFP and HCG [27]	Multiple FDG-avid residual masses [53]
Elevated pre-chemotherapy LDH [27] ^*3^	Post-chemotherapy nodal size [24]
Small residual mass (< 10–20 mm in small transverse diameter) [27] ^*2^	Supra-diaphragmatic lymph nodes and/or visceral metastasis [6]
Marked residual mass reduction (> 70–90%) [27]	Prior history of relapse [25]
< 10% residual viable cells in the PC-RPLND specimen [16]	Late-onset relapse (i.e., > 2 years) following chemotherapy [25]

^*1^ Predictors of teratoma in post-chemotherapy retroperitoneal lymph node dissection (PC-RPLND) specimen includes presence of teratoma and yolk sac tumor in the orchiectomy specimens [11]. Absence of teratoma in the orchiectomy specimen does not exclude the presence of teratoma in PC-RPLND [24, 25]

^*2^ Predictors of necrosis in PC-RPLND specimen includes presence of seminomatous and absence of teratomatous elements in the primary tumor, normal pre-chemotherapy beta-hCG and AFP levels, small pre-chemotherapy (cutoff: <2 cm) or post-chemotherapy (cutoff: ≤1 cm) lymph nodes and >50% mass size reduction following chemotherapy [26, 30]

^*3^ The International Germ Cell Cancer Collaborative Group prognostic classification also considers elevated LDH levels in the poor prognostic group [9]

Some authors have suggested that the incorporation of non-invasive imaging modalities, such as ^18^F-FDG PET/CT, into the management algorithm may allow better prediction of viable residual tumors and, thus better risk stratification in this setting [4]. However, the NCCN guideline [32] recommends abdominopelvic ceCT, MRI, and CXR as modalities for imaging first- and second-line chemotherapy patients and routine follow-up cases, which is also consistent with other guidelines (e.g., ESMO, SWENOTECA) [33–35]. The NCCN guidelines currently recommend against the routine use of ^18^F-FDG PET/CT while considering its possible usefulness for surveillance of patients in the post-chemotherapy status [32].

### Why ^18^F-FDG PET/CT was not so successful?

**Fig. 1:**
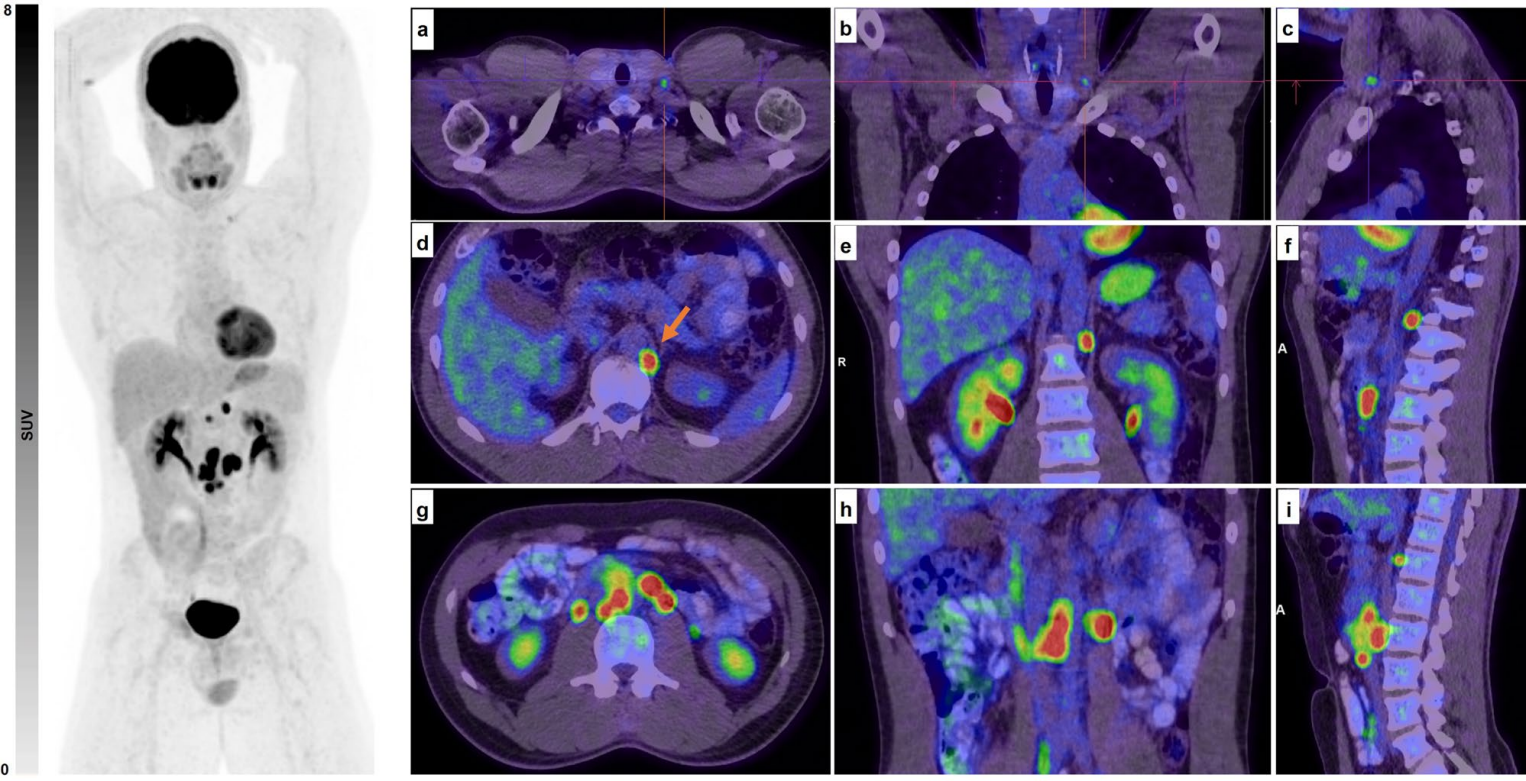
A 32-year-old male with non-seminomatous testicular cancer, initially treated with orchidectomy, presented with suspicious para-aortic lymph nodes on CT and underwent an FDG PET scan. The images reveal a subcentimetre left supraclavicular lymph node (SUVmax 3.5; **images a-c**,** crosshairs**), bilateral intensely FDG-avid retrocrural lymph nodes (SUVmax 8 on the right and 12 on the left; **images d-f**,** red arrow**), and bilateral para-aortic lymphadenopathy extending from the axial level of L1 to L2/L3 on the right (SUVmax 21) and from the axial level of L1/L2 to L2/L3 on the left (SUVmax 25; **images g-I**). A subsequent biopsy of the left supraclavicular node confirmed metastatic involvement. Retroperitoneal lymph node dissection also confirmed multifocal retroperitoneal nodal metastases

The utility of ^18^F-FDG PET/CT in patients with NSGCT has been a topic of considerable debate, with views ranging from some to no benefit [36–38]. Viable tumors have significantly higher FDG uptake (Fig. 1) as compared to the generally low FDG uptake in necrosis, fibrosis, or teratoma [39]. Also, a negative ^18^F-FDG PET/CT scan has been linked with increased overall survival [40]. ^18^F-FDG PET/CT has also been investigated in the context of relapse following definitive NSGCT treatment. It has also been shown that the levels of tumor markers (i.e., LDH, AFP, and hCG) have a significantly positive correlation with the ^18^F-FDG uptake [41].

**Fig. 2:**
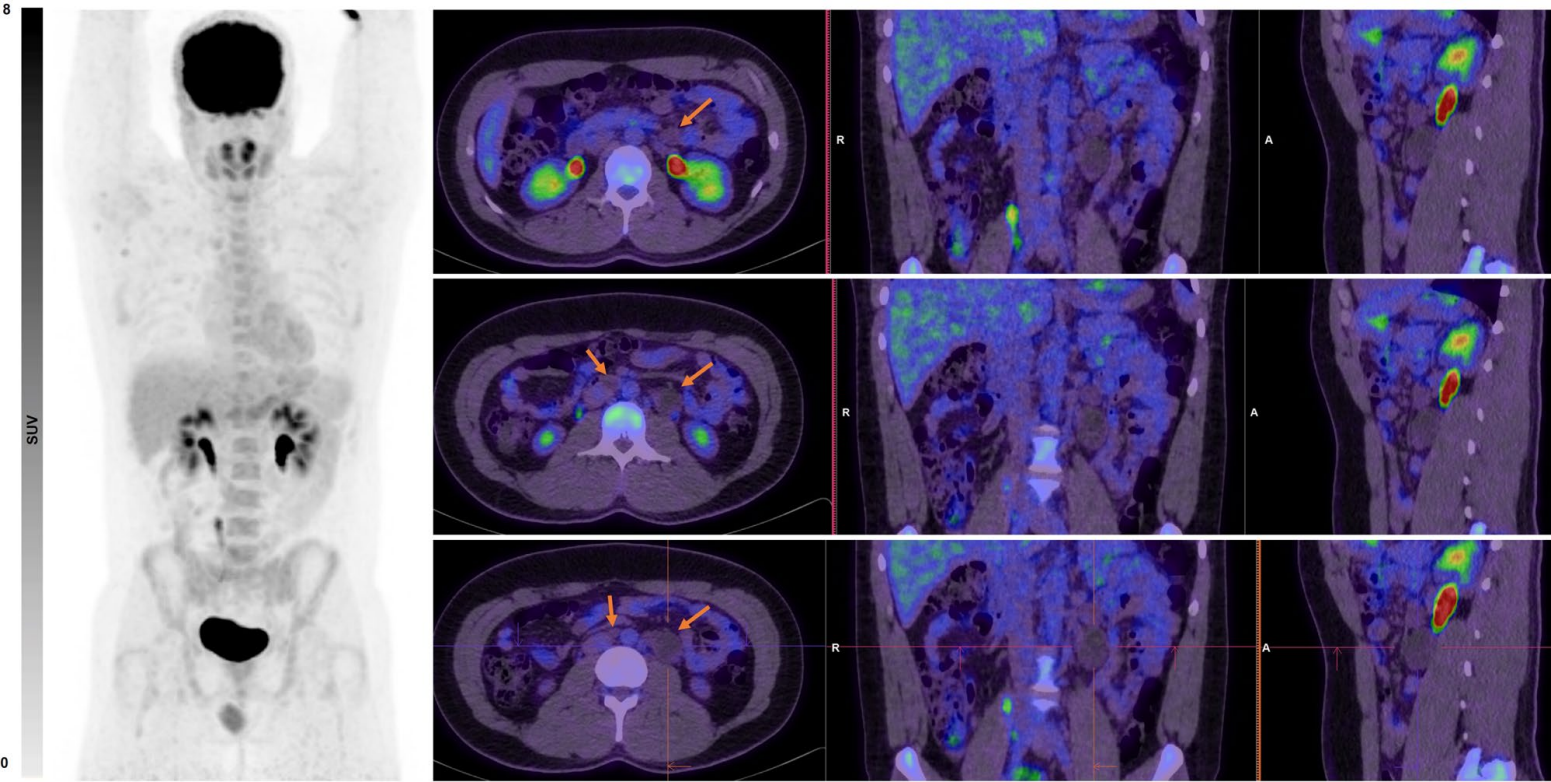
A 30-year-old male with a non-seminoma germ cell tumor, previously treated with a left orchidectomy. The PET scan demonstrates multiple low-density nodal lesions in the retroperitoneum, including at the aortocaval and left para-aortic stations (**red arrows**), with no increased FDG uptake. The patient subsequently underwent retroperitoneal lymph node dissection, which revealed multiple nodal metastases

**Fig. 3:**
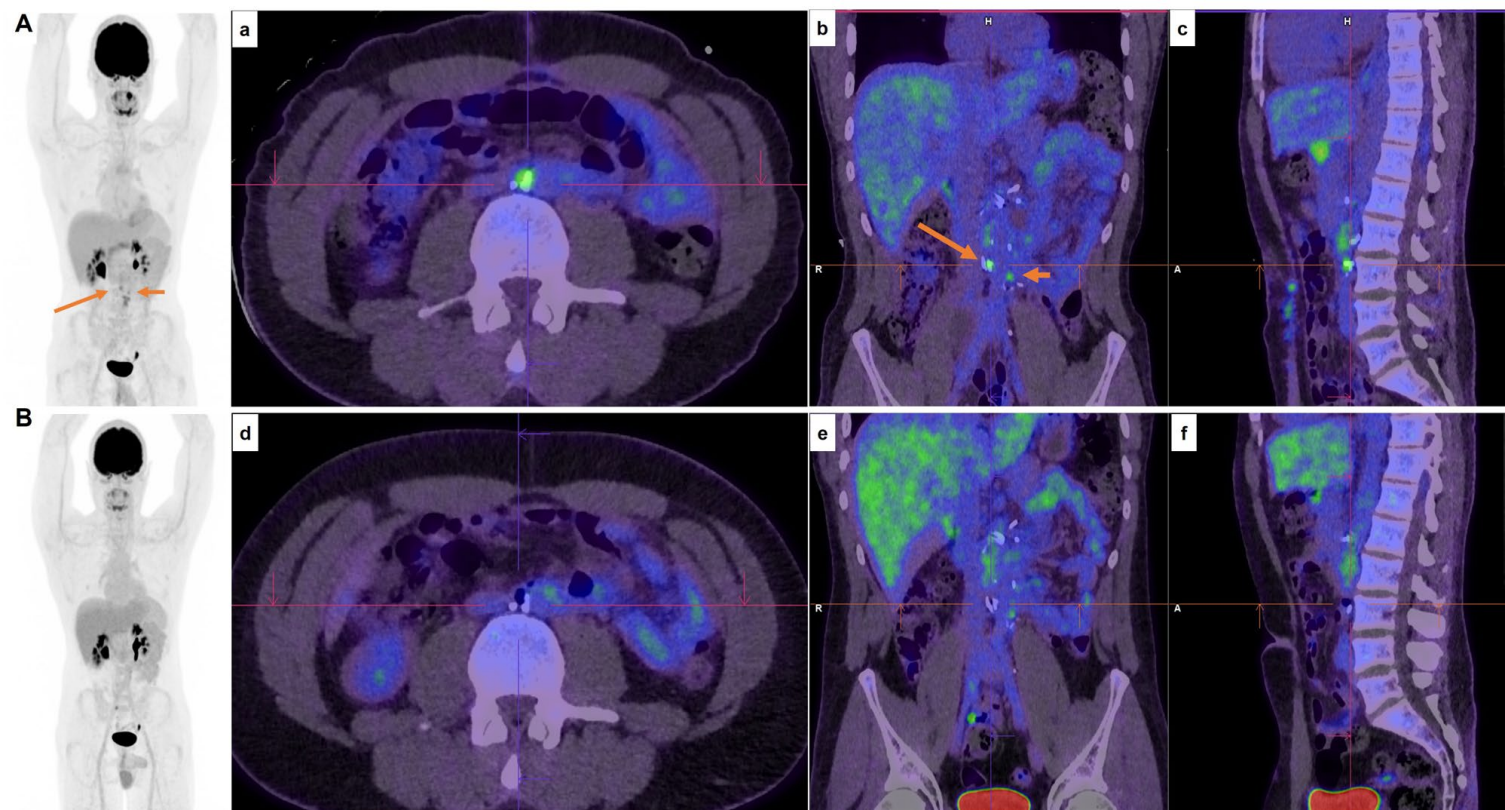
A 33-year-old male with a history of non-seminoma germ cell tumor, previously treated with left orchidectomy, chemotherapy, and retroperitoneal lymph node dissection for bulky para-aortic lymph node metastases, presents for a progress assessment. The PET scan **(Image A)** demonstrates mild foci of uptake around the aortocaval (SUV max 3.9) and left para-aortic (SUV max 3.3) regions at the level of L3, adjacent to surgical clips (**red arrows**, **a-c**), which were reported as indeterminate (either post-surgical inflammatory changes or residual disease). Twelve months later, a follow-up PET scan (**Image B**) showed an interval reduction in the intensity of retroperitoneal foci of uptake (**d-f**), consistent with resolving post-operative inflammatory changes

Contrary to the studies mentioned above, in one study, ^18^F-FDG PET was even inferior to CT for differentiation of necrosis/fibrosis from teratoma [42] (Fig. 2), which is considered the main disadvantage of ^18^F-FDG PET/CT [43–46]. Moreover, ^18^F-FDG PET/CT may falsely show extensive uptake in post-chemotherapy inflammatory changes [47], especially if imaged early post-treatment [12] (Fig. 3). Furthermore, it will miss small (i.e., < 5–10 mm) lesions [48, 49], leading to high relapse rates among the PET-negative patients [50]. Therefore, its application for routine staging of NSGCT is discouraged since it will not have a clear added value to the standard ceCT and will not alter the treatment management [7, 23, 51, 52]. Also, for prediction of response to chemotherapy, ^18^F-FDG PET/CT has not been shown to be superior to ceCT or serum tumor markers, although being a strong predictor of pathologic viable disease [37, 53, 54].

**Fig. 4:**
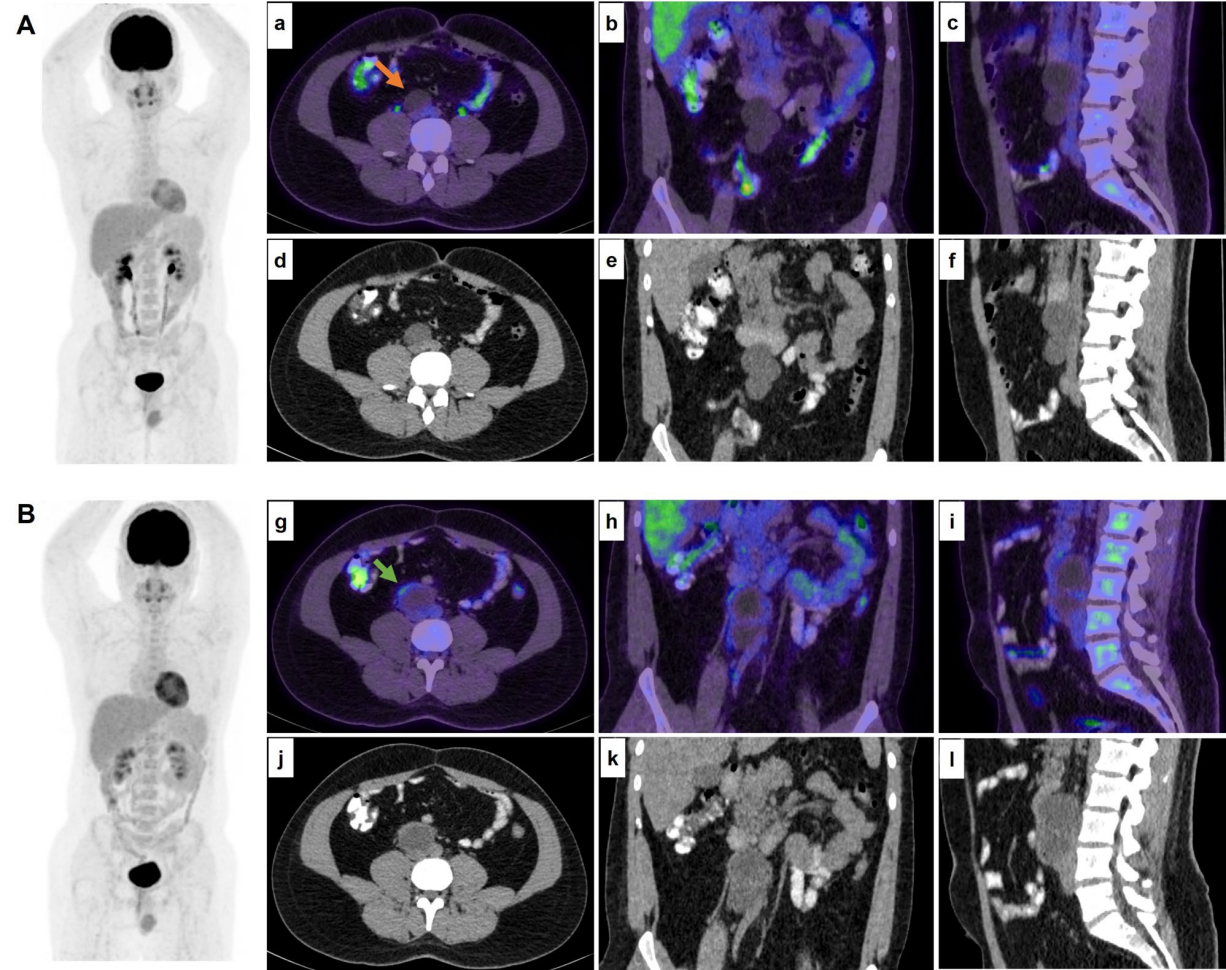
A 23-year-old male with non-seminomatous testicular cancer, treated with orchidectomy followed by chemotherapy, presented with suspicious retroperitoneal lymphadenopathy on CT and underwent an FDG PET scan (**Image A**). The axial (**a**,** d**), coronal (**b**,** e**), and sagittal (**c**,** f**) views show enlarged hypoattenuating retroperitoneal lesions with no metabolic activity (**red arrow**). Serial CT scans demonstrated ongoing enlargement of these lesions. Ten months later, a follow-up PET scan (**Image B**) revealed further enlargement of the hypoattenuating retroperitoneal lesions with interval development of peripheral metabolic activity (**images g-l**,** green arow**), highly suggestive of nodal metastases. Subsequent nodal dissection confirmed the presence of nodal metastases on histopathology

In one study in NSGCT patients examined 85 residual lesions, with 32 (38%) showing increased tracer uptake, resulting in a sensitivity of 59%, specificity of 92%, NPV of 62%, and PPV of 91% [53]. Therefore, ^18^F-FDG PET positivity may be clinically relevant in evaluation of residual masses. In another study, quantitative ^18^F-FDG PET analyses indicated significant differences between mature teratoma and necrosis or scar tissue, supporting its use for evaluating residual lesions post-chemotherapy [55]. Additionally, another study found that an SUV greater than 5 is more likely to be linked to viable GCT than necrosis, fibrosis, or mature teratoma [36]. However, the overall diagnostic benefit of ^18^F-FDG PET/CT over traditional markers and CT scans for suspected NSGCT recurrence remains uncertain (Fig. 4), though patients with elevated tumor markers with equivocal CT findings might benefit from ^18^F-FDG PET/CT [56, 57].

In conclusion, in the era of “forget the PET” approach, ^18^F-FDG PET/CT may infrequently be requested for rising tumor markers with normal ceCT and those with equivocal ceCT findings [58, 59]. Kinetic analysis may improve its diagnostic performance but is not performed in routine clinical practice, and has not been fully validated in large prospective studies [55].

## Teratoma imaging with other radiopharmaceuticals: a land of failures

The multi-layered embryologic origin of teratoma sometimes contains immature neuro-ectodermal elements, which may eventually trap radioiodine or radiotracers targeting the somatostatin-receptors [60, 61]. Multiple research groups have explored the added value of other PET radiopharmaceuticals, most of which were not very successful (Table 3). Perhaps the best one was imaging with radiopharmaceuticals targeting integrins. Yet, none of these radiotracers find their way into clinical practice. A more in-depth review of the experience gained by each imaging modality is discussed as follows.

**Table 3:** Teratoma imaging with non-^18^F-FDG radiotracers ^*1^

Radiotracer class [Name of the radiotracer]	Research aim	Sample size	Study type	Encouraging results?	First author (Reference)
Integrins - α_v_β_3_ [^99m^Tc-HYNIC-RGD]	Mature teratoma vs. necrosis	20 rats	Preclinical animal study	Yes, for αvβ3 imaging No, for FDG	Aide [110]
Integrins - α_v_β_3_ [^99m^Tc-3PRGD2]	Detection of hiPSC-derived teratoma	4 rats	Preclinical animal study	Yes, for αvβ3 imaging No, for FDG	Li [67]
Integrins α_v_β_3_ [^64^Cu-DOTA-RGD4]	In vivo visualization of teratoma formation	12 rats	Preclinical animal study	Yes, for αvβ3 imaging No, for FDG and FLT	Cao [111]
Integrins - α_v_β_3_ [^99m^Tc-HYNIC-RGD]	Biodistribution and imaging of α_v_β_3_-negative and positive tumors	4 rats	Preclinical animal study	Equivocal, no definite conclusion regarding the main reason for integrin uptake (i.e., vasculature vs. cellular expression)	Bohn [112]
Radioiodine - ^124^I	In vivo visualization of teratoma formation	9 rats	Preclinical animal study	Yes, highly specific; Tracer uptake correlated with teratoma weight and washed out with perchlorate	Lehner [113]
^18^F-FLT [39-deoxy-39–18 F-fluorothymidine]	Added value of FLT on top of FDG PET	11 patients (2 teratoma cases)	Case series	No, FDG and FLT were both falsely negative.	Pfannenberg [63]
^67^Ga-Citrate	Detection rates in various GU malignancies	11 teratoma cases (16 lesions)	Retrospective clinical cases	No, low detection rate (25%)	Sauerbrunn [72]
PSMA: - ^68^Ga-PSMA/^177^Lu-PSMA	Single dose of ^177^Lu-PSMA RLT in a case of refractory mixed GCT	1 patient	Case report	No, rise in AFP and evidence of tumoral progression in the hepatic lesion despite sufficient PSMA uptake	Simsek [66] ^*2^
FAPi: - ^68^Ga-FAPi-04	Comparison with FDG uptake	1 patient	Case report	Equivocal, FAPI (moderate uptake) vs. FDG (low uptake)	Kaplan [1] ^*3^
^18^F-Fluciclovine	Detection of residual disease/teratoma	10 patients (5 teratoma cases)	Case series	No, low detection rate (40%) in the teratoma subgroup	Woldu [64]

^*1^ Staging with somatostatin receptor scintigraphy and mIBG has been attempted for teratoma cases associated with TNET. Due to its rarity, the added value of these imaging modalities to conventional imaging is not yet demonstrated [114, 115]. Moreover, there are a few case reports/series for incidental detection of non-seminomatous GCT with ^99m^Tc-pertechnetate and ^99m^Tc-MDP or non-visualization of teratocarcinoma using ^201^Tl [71–73]

^*2^ Tried in the light of prior human tissue studies [65]

^*3^ Phase I studies are ongoing [116]

GCT = Germ cell tumor; GU = Genitourinary; hiPSC = human-induced pluripotent stem cell; RLT = Radioligand therapy; TNET = Testicular neuroendocrine tumor

## PET tracers

### ^11^C-tyrosine PET

A study showed that ^11^C-tyrosine is not suited to visualize the apparently slowly proliferating NSGCT or to determine the nature of a residual mass after chemotherapy [62].

### ^18^F- fluorothymidine (^18^F-FLT)

In a small series of 11 patients (10 NSGCT, 1 seminoma) with metastatic NSGCTs, Pfannenberg et al. compared the diagnostic value of ^18^F-FLT, which measures tumor cell proliferation, with ^18^F-FDG PET/CT. Despite the lower incidence of false-positive results with ^18^F-FLT PET than with ^18^F-FDG PET, the low negative predictive value of ^18^F-FDG PET could not be improved by the application of the ^18^F-FLT (60% and 50%, respectively). Therefore, PET-negative residual masses after chemotherapy of metastatic NSGCT still require resection. The low sensitivity of ^18^F-FLT PET/CT for the detection of viable residual tumors in this study may be related to the lower tissue uptake of ^18^F-FLT than of ^18^F-FDG in GCTs. Positive results on ^18^F-FDG PET after chemotherapy correlated strongly with the presence of viable tumors. For prediction of response after completion of chemotherapy, the final PET/CT scan, whether performed using ^18^F-FDG or using ^18^F-FLT, cannot be replaced by early response evaluation [63].

### ^18^F-fluciclovine

The potential use of ^18^F-fluciclovine for molecular imaging of NSGCTs was evaluated in a small prospective study, which revealed poor sensitivity and specificity in detecting teratoma from fibrosis/necrosis in patients with residual masses undergoing PC-RPLND. Half of the negative ^18^F-fluciclovine PET/CT cases were found to have residual disease/teratoma following surgery. The low utility of ^18^F-fluciclovine PET/CT in guiding the management of NSGCT post-chemotherapy was evident, with sensitivity and specificity rates at 29% and 33%, respectively [64].

## Prostate-Specific Membrane Antigen (PSMA)

Prior human tissue studies have shown the expression of PSMA in some cases of NSGCT [65]. A case report of metastatic mixed (immature teratoma and yolk sac carcinoma) testicular GCT with acceptable tumor-to-background ratio was treated with the therapeutic counterpart of PSMA, namely ^177^Lu-PSMA, was not successful [66].

## Integrins

Since their discovery in 2006, induced pluripotent stem cells (iPSCs) have gained increasing interest in tissue regeneration and transplantation therapies. However, teratoma formation after iPSC transplantation is one of the most serious drawbacks of this procedure. In a study, it was investigated whether human iPSC-derived teratomas could be detected by an integrin-targeting agent, ^99m^Tc-PEG4-E[PEG4-c(RGDfK)]2 (^99m^Tc-3PRGD2). Gamma camera imaging with ^99m^Tc-3PRGD2 may be a promising approach for the non-invasive monitoring of tumorigenicity after human iPSCs transplantation [67]. Unfortunately, these preclinical observations were never explored on human subjects.

### Non-PET Tracers

SPECT/CT’s spatial resolution is challenged over PET/CT, particularly in small lesions that are not always metabolically active, including NSGCTs [68]. This gap can be bridged with advanced quantification and reconstruction techniques and multi-pinhole collimators focusing on gamma rays [69]. PET facilities are preferred over SPECT in regions where both options are available; however, SPECT remains cost-effective in specific clinical applications and resource-limited countries [70, 71]. The results of teratoma imaging utilizing SPECT are unsatisfactory [71–80]. Small case series and case reports reported non-visualization of teratocarcinoma using Tl-201 [71] or incidental detection of NSGCTs using 99mTc-MDP due to ossification and cartilage tissue in teratoma [72], and 99mTc-pertechnetate due to increased flow in tumoral tissue [73].

## Gallium-67

Traditionally, ^67^Ga scintigraphy was considered valuable in assessing the intra-abdominal spread of malignant tumors of the testes. However, it appeared that metastatic tumors of the embryonal-cell and seminoma type, compared to teratoma, are more readily detectable by gallium-67 scanning [72]. Although its application for imaging of NSGCT were disappointing and discontinued [73, 74], its utility for staging in seminoma also became obsolete after the introduction of ^18^F-FDG PET/CT [75].

## Radiolabeled antibody

Radioimmunodetection captures tumor-specific or tumor-associated markers by preferentially accumulating tumor-specific antisera in tumoral tissues. Murine teratocarcinomas were localized using external gamma-ray scintigraphy with ^131^I-labeled monoclonal antibodies. By removing background radioactivity from the control monoclonal antibody 123 of the same immunoglobulin class, detection was enhanced [76]. Javadpour et al. utilized ^131^I-labeled antibodies targeting tumor-associated antigens in testicular cancer to identify occult disease [77]. The limited sensitivity of this approach in identifying lesions under 2 × 2 cm and interference from background radioactivity limit its practical applicability. Epenetos et al. investigated placental alkaline phosphatase-targeting indium-111 monoclonal antibodies. Their study showed improved ovarian, cervical, and testicular cancer diagnosis. However, there are still ongoing issues regarding the pharmacokinetics and immunogenicity of antibodies, despite the positive outcomes shown [78].

### Potential of imaging teratoma with novel radiotracers

#### Fibroblast activation protein inhibitor (FAPi)

FAP-targeting PET tracers have been extensively studied in both malignant and non-malignant entities [79, 80]. FAPi ligands may have a complementary role in detecting metastatic lymph nodes, especially if coupled with ^18^F-FDG PET imaging in various cancers [81]. In addition, FAPi PET imaging has been shown to be able to detect fibrotic tissue in various scenarios (e.g., post-chemotherapy fibrosis in GI malignancies, idiopathic retroperitoneal fibrosis, and various non-malignant fibrotic pathologies) [79, 82]. However, to the best of our knowledge, till today, there has been no published comprehensive paper on FAPi PET tracers in NSGCTs.

Regarding FAP application in detecting teratoma, there is insufficient data in the literature. Xi et al., in a study conducted to compare ^68^Ga-FAPi-04 PET/MR and ^18^F-FDG PET/CT in ovarian tumors in 2023, and reported that of all the included cases, two patients had teratoma (one considered benign pathology and the latter borderline) [83]. In a study comparing ^68^Ga-FAP-2286 PET/CT head-to-head with ^18^F-FDG PET/CT in various malignancies, one case of metastatic yolk sac germ cell tumor was evaluated and showed better LN detection performance of FAPi ligand over ^18^F-FDG with a better target-to-background ratio (separate values are not reported). Visually, in comparison to [^68^Ga]Ga-FAP-46, this new FAP ligand seems to have a higher uptake [84].

There are two additional papers that each report one case of GCT imaged with FAPi radiotracers. Dai et al. [85]. reported a rare case of extragonadal yolk-sac tumor in which ^68^Ga-FAPI PET/MR outperformed ^18^F-FDG PET/CT in the detection of the cranial lesion. The other case report looked at ^68^Ga-FAPi-04 PET/CT and ^18^F-FDG PET/CT in a person who had mixed testicular GCT that was 65% post-pubertal teratoma, 25% yolk sac and 10% seminoma. The retroperitoneal and lung nodules showed a slight uptake of FDG. Meanwhile, FAPi imaging revealed a mild-moderate uptake in the affected lesions (SUV_max_ of 3.9) [1].

### CXCR4

CXCR4 is a seven transmembrane domain G protein-coupled receptor (GPCR) that contributes to chemotaxis, invasion, angiogenesis, aggressiveness, tumor progression, proliferation, and metastasis [86]. The CXCR4 ligand is frequently overexpressed in various types of cancer [87]. The CXCL12/CXCR4 pathway has a confirmed and significant role in the adult human testis microenvironment and is also expressed in gonadal and extragonadal GCTs [88, 89]. Yet, in all the publications concerning cellular studies, there is no clinical data to support this hypothesis.

### Ghrelin

Testicular tumors differentially express the Ghrelin receptor, a GPCR involved in growth hormone secretion and food intake. In order to enhance in vivo stability and incorporate the Fluorine-18 isotope for PET imaging, the ghrelin ligand has recently undergone some modifications. This novel PET agent has been shown to have a high affinity for the ghrelin receptor in biochemical and preclinical studies [90, 91]. For now, there is no available clinical (in humans) data regarding Ghrelin receptor imaging in NSGCT.

### Other solutions beyond nuclear medicine approaches

#### Magnetic Resonance Imaging

Advancements in the field of MRI in patients with testicular cancer are threefold. First, follow-up whole-body MRI may be employed in the future in lieu of ceCT due to concerns regarding radiation exposure and in the light of positive non-inferiority trials recently published in this regard [92, 93]. Second, new MRI sequences, namely T_1_-Dixon and T_2_-BLADE, have been shown to propose better performance in detecting retroperitoneal metastasis and were better than DWI-MRI in a prospective study [94]. Third, lymphotropic nanoparticle MRI (LNMRI) utilizes nanoparticles that aggregate with a mixed signal within pathological nodal tissue [95]. Its use is superior to conventional MRI, according to meta-analyses, in terms of both sensitivity (88% vs. 63%) and specificity (96% vs. 95%) [96, 97]. Harisinghani et al. [97] conducted a pilot trial of LNMRI to detect occult metastases in 18 men with testicular cancer. LNMRI had improved sensitivity (88% vs. 71%) and specificity (92% vs. 68%) compared to MRI or CT size criteria among these patients. Likewise, LNMRI was 100% sensitive in detecting positive lymph nodes less than 10 mm, that would not have been considered suspicious on conventional imaging. However, the lengthy duration (24–36 h) between nanoparticle injection and MRI, along with the requirement for an experienced radiologist to accurately interpret the images, restricts its adoption [54].

### The potential role of radiomics

Literature regarding the added value of radiomics to differentiate necrosis/fibrosis from teratoma is emerging, with some studies showing encouraging results [98, 99]. As of now, these studies are yet insufficient to precisely select patients for PC-RPLND to prevent over-treatment [100, 101], inconclusive [99–103] and sometimes controversial [98, 100, 104–106]. Moreover, non-automated approaches for delineation of the regions of interest are time consuming and not repeatable, limiting its practicality in daily practice [107].

### microRNAs: a potential target for imaging?

A recent study used molecular analysis to explore a non-imaging method to differentiate between teratoma, viable GCT, and necrosis post-chemotherapy [108]. This approach identified AGR2 and KRT19 as key proteins significantly overexpressed in teratoma compared to necrosis at both microRNA and protein levels.

This approach involved classifying 48 patients into three groups: those with teratoma, those with viable GCT, and those with necrosis. Using a microdissection technique, they precisely isolated representative areas of each tissue type within the lymph nodes [108].

From a nuclear medicine perspective, if these proteins are tagged with a PET tracer, it would shed light on the precise diagnosis in this gray area.

## Conclusion

Imaging of NSGCT remains challenging, and while ^18^F-FDG PET imaging has limitations, in a few selected scenarios is still able to contribute to clinical management decisions. The experiences with non-^18^F-FDG radiotracers have not yet identified a compelling radiotracer for use in this clinical scenario. For now, the complementary benefits of different imaging techniques could be a reasonable approach. The introduction of miRNAs is speculated to revolutionize the field, which are great candidates for future targets to be radiolabeled for imaging NSGCT. The evolving role of radiomics, which remains inconclusive in the field of NSGCT, is still in its infancy but may eventually become a part of routine practice.

## Data Availability

No datasets were generated or analysed during the current study.

## Abbreviations

ceCT: contrast-enhanced CT
FAPi: Fibroblast activation protein inhibitor
GPCR: G protein-coupled receptor
iPSCs: induced pluripotent stem cells
LNMRI: lymphotropic nanoparticle MRI
NSGCT: non-seminomatous germ cell tumors
PC-RPLND: Post-chemotherapy retroperitoneal lymph node dissection
PSMA: Prostate-Specific Membrane Antigen
RAID: radioimmunodetection
